# Neurosarcoidosis Limited to the Central Nervous System and Spleen, Presenting with Episodic Nonfluent Aphasia

**DOI:** 10.31662/jmaj.2025-0067

**Published:** 2025-06-27

**Authors:** Satoka Yano, Takashi Matsukawa, Kensho Sumi, Reo Yoshioka, Yusuke Baba, Hirotaka Maekawa, Masashi Hamada, Wataru Satake, Masako Ikemura, Tatsushi Toda

**Affiliations:** 1 Department of Neurology, Graduate School of Medicine, The University of Tokyo, Tokyo, Japan; 2 Department of Pathology, Graduate School of Medicine, The University of Tokyo, Tokyo, Japan

**Keywords:** neurosarcoidosis, nonfluent aphasia, splenomegaly, splenic biopsy, epithelioid cell granuloma

A 19-year-old right-handed previously healthy male presented with episodic, nonfluent aphasia lasting approximately 1 hour every few days for 4 months. Brain magnetic resonance imaging (MRI) revealed multiple hyperintense lesions in the superior temporal gyrus and supramarginal gyrus in fluid-attenuated inversion recovery images with linear contrast enhancement along the medullary veins (Figure 1A and B). Dilated medullary veins and multiple microbleeds were also noted in susceptibility-weighted imaging (Figure 1C). Abdominal MRI revealed mild splenomegaly and a heterogeneously enhanced lesion with variable contrast enhancement during the portal venous phase (Figure 1D). ^18^F-fluorodeoxyglucose positron emission tomography/computed tomography showed abnormal uptake corresponding to this splenic region, with a maximal standardized uptake value of 3.1 (Figure 1E). Ultrasound-guided splenic biopsy confirmed clustered epithelioid cell granulomas without caseous necrosis (Figure 1F), leading to the diagnosis of probable neurosarcoidosis. Comprehensive evaluations revealed no evidence of abnormalities in any other organs, including those in the pulmonary, cutaneous, cardiac, or ophthalmologic system.

**Figure 1.**
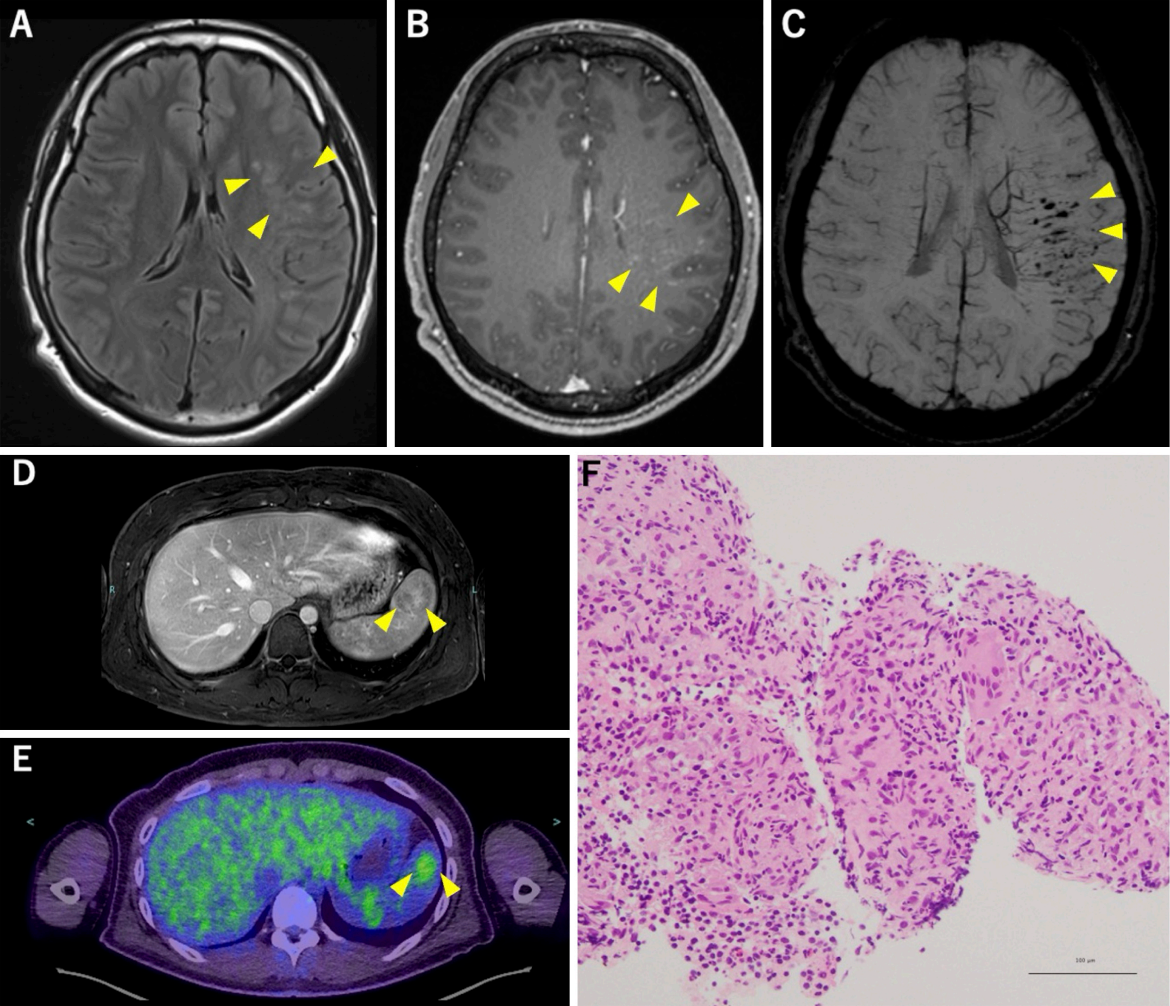
Brain MRI reveals hyperintense lesions in the left inferior frontal gyrus, superior temporal gyrus, and supramarginal gyrus on axial FLAIR (A, yellow arrowheads), and gadolinium-enhanced T1-weighted imaging shows linear contrast enhancement along medullary veins (B, yellow arrowheads). Susceptibility-weighted imaging reveals dilated medullary veins and multiple hypointense areas indicative of microbleeds (C, yellow arrowheads). Abdominal MRI reveals mild splenomegaly and a focal lesion with heterogeneous contrast enhancement during portal venous phase (D, yellow arrowheads), and ^18^F-FDG-PET/CT shows abnormal uptake corresponding to this splenic region, with a an SUV max of 3.1 (E, yellow arrowheads). A splenic biopsy confirms clusters of epithelioid cells without caseous necrosis (F). ^18^F-FDG-PET/CT: ^18^F-fluorodeoxyglucose positron emission tomography/computed tomography; FLAIR: fluid-attenuated inversion recovery; MRI: magnetic resonance imaging; SUV max: maximal standardized uptake value.

Seizures in neurosarcoidosis ^(1)^ may result from brain parenchymal, meningeal lesions, or granuloma-associated small vessel vasculitis ^(2)^. In sarcoidosis, cases without pulmonary involvement are uncommon (8%) ^(3)^, and reports of lesions confined solely to the central nervous system (CNS) and spleen are exceedingly rare. However, considering the challenges associated with obtaining CNS biopsies, it is noteworthy that splenic biopsy aids in diagnosis.

## Article Information

### Conflicts of Interest

None

### Author Contributions

Satoka Yano and Takashi Matsukawa acquired data and drafted the manuscript. Kensho Sumi, Reo Yoshioka, Yusuke Baba, Hirotaka Maekawa, Masashi Hamada, Wataru Satake, Masako Ikemura, and Tatsushi Toda edited and approved the final manuscript.

### Informed Consent

Informed consent was obtained from the patient.

### Approval by Institutional Review Board (IRB)

IRB approval was not required for this study.
